# Comparison of therapeutic effects of mesenchymal stem cells derived from superficial and deep subcutaneous adipose tissues

**DOI:** 10.1186/s13287-023-03350-3

**Published:** 2023-05-04

**Authors:** Naoki Ishiuchi, Ayumu Nakashima, Satoshi Maeda, Yoshie Miura, Kisho Miyasako, Kensuke Sasaki, Toshio Uchiki, Ayano Sasaki, Shogo Nagamatsu, Naoki Nakao, Masataka Nagao, Takao Masaki

**Affiliations:** 1 Department of Nephrology, Hiroshima University Hospital, 1-2-3 Kasumi, Minami-ku, Hiroshima, 734-8551 Japan; 2 Center for Cause of Death Investigation Research, Graduate School of Biomedical & Health Sciences, Hiroshima University, 1-2-3 Kasumi, Minami-ku, Hiroshima, 734-8553 Japan; 3 Department of Forensic Medicine, Graduate School of Biomedical & Health Sciences, Hiroshima University, 1-2-3 Kasumi, Minami-ku, Hiroshima, 734-8553 Japan; 4 Department of Stem Cell Biology and Medicine, Graduate School of Biomedical & Health Sciences, Hiroshima University, 1-2-3 Kasumi, Minami-ku, Hiroshima, 734-8553 Japan; 5 TWOCELLS Company, Limited, 16-35 Hijiyama-honmachi, Minami-ku, Hiroshima, 732-0816 Japan; 6 Department of Plastic and Reconstructive Surgery, Hiroshima University Hospital, 1-2-3 Kasumi, Minami-ku, Hiroshima, 734-8551 Japan

**Keywords:** Mesenchymal stem cells, Superficial subcutaneous adipose tissue, Deep subcutaneous adipose tissue, Intravascular mesenchymal stem cell therapy

## Abstract

**Background:**

Fibrosis is a common histological feature in the process from chronic organ injury to organ failure. Chronic tissue injury causes inflammatory cell infiltration into the injured tissue. The persistence of this inflammatory cell infiltration leads to fibrosis and organ failure. Adipose-derived mesenchymal stem cells (ASCs) have received much attention as a regenerative therapeutic tool to prevent progression from organ injury to failure. Subcutaneous abdominal adipose tissue is divided into superficial and deep layers by a superficial fascia. Adipose tissue easily collected by liposuction is usually obtained from a deep layer, so ASCs derived from a deep layer are generally used for regenerative medicine. However, no research has been conducted to investigate differences in the therapeutic effects of ASCs from the superficial and deep layers (Sup-ASCs and Deep-ASCs, respectively). Therefore, we compared the therapeutic potencies of Sup-ASCs and Deep-ASCs.

**Methods:**

ASCs were isolated from superficial and deep subcutaneous abdominal adipose tissues collected from patients who underwent breast reconstruction. We first compared cell characteristics, such as morphology, cell proliferation, cell surface markers, adipogenic and osteogenic differentiation, cell senescence markers, and expression of coagulation and anticoagulant factors between Sup-ASCs and Deep-ASCs. Furthermore, we compared their ability to promote polarization of M2 macrophages and to inhibit transforming growth factor (TGF)-β/Smad signaling using THP-1 cells and TGF-β1 stimulated HK-2 cells incubated with conditioned media from Sup-ASCs or Deep-ASCs. In in vivo experiments, after renal ischemia–reperfusion injury (IRI) procedure, Sup-ASCs or Deep-ASCs were injected through the abdominal aorta. At 21 days post-injection, the rats were sacrificed and their left kidneys were collected to evaluate fibrosis. Finally, we performed RNA-sequencing analysis of Sup-ASCs and Deep-ASCs.

**Results:**

Sup-ASCs had greater proliferation and adipogenic differentiation compared with Deep-ASCs, whereas both ASC types had similar morphology, cell surface markers, senescence markers, and expression of coagulation and anticoagulant factors. Conditioned media from Sup-ASCs and Deep-ASCs equally promoted polarization of M2 macrophages and suppressed TGF-β/Smad signaling. Moreover, administration of Sup-ASCs and Deep-ASCs equally ameliorated renal fibrosis induced by IRI in rats. RNA-sequencing analysis revealed no significant difference in the expression of genes involved in anti-inflammatory and anti-fibrotic effects between Sup-ASCs and Deep-ASCs.

**Conclusions:**

These results indicate that both Sup-ASCs and Deep-ASCs can be used effectively and safely as an intravascular ASC therapy for organ injury.

**Supplementary Information:**

The online version contains supplementary material available at 10.1186/s13287-023-03350-3.

## Background

Fibrosis is a common histological feature in the process from chronic organ injury to organ failure [1–3], including renal failure [1, 4], cirrhosis [1, 5], pulmonary fibrosis [1, 6], and heart failure [1, 7]. Chronic tissue injury caused by lifestyle-related diseases, such as diabetes, hypertension, and obesity, induces the release of damage-associated molecular patterns, causing inflammatory cell infiltration into the injured tissue [8, 9]. The persistence of this inflammatory cell infiltration activates fibroblasts, leading to fibrosis and organ failure [10, 11]. Although various drugs have been developed to inhibit fibrotic progression, the beneficial effects of these drugs are moderate, and many patients eventually develop organ failure. Many studies have reported that mesenchymal stem cells (MSCs) exert anti-inflammatory and anti-fibrotic effects via their paracrine actions [12, 13]. Thus, MSCs are expected to be a new therapeutic tool to prevent progression from organ injury to failure [14–16].

**Fig. 1:**
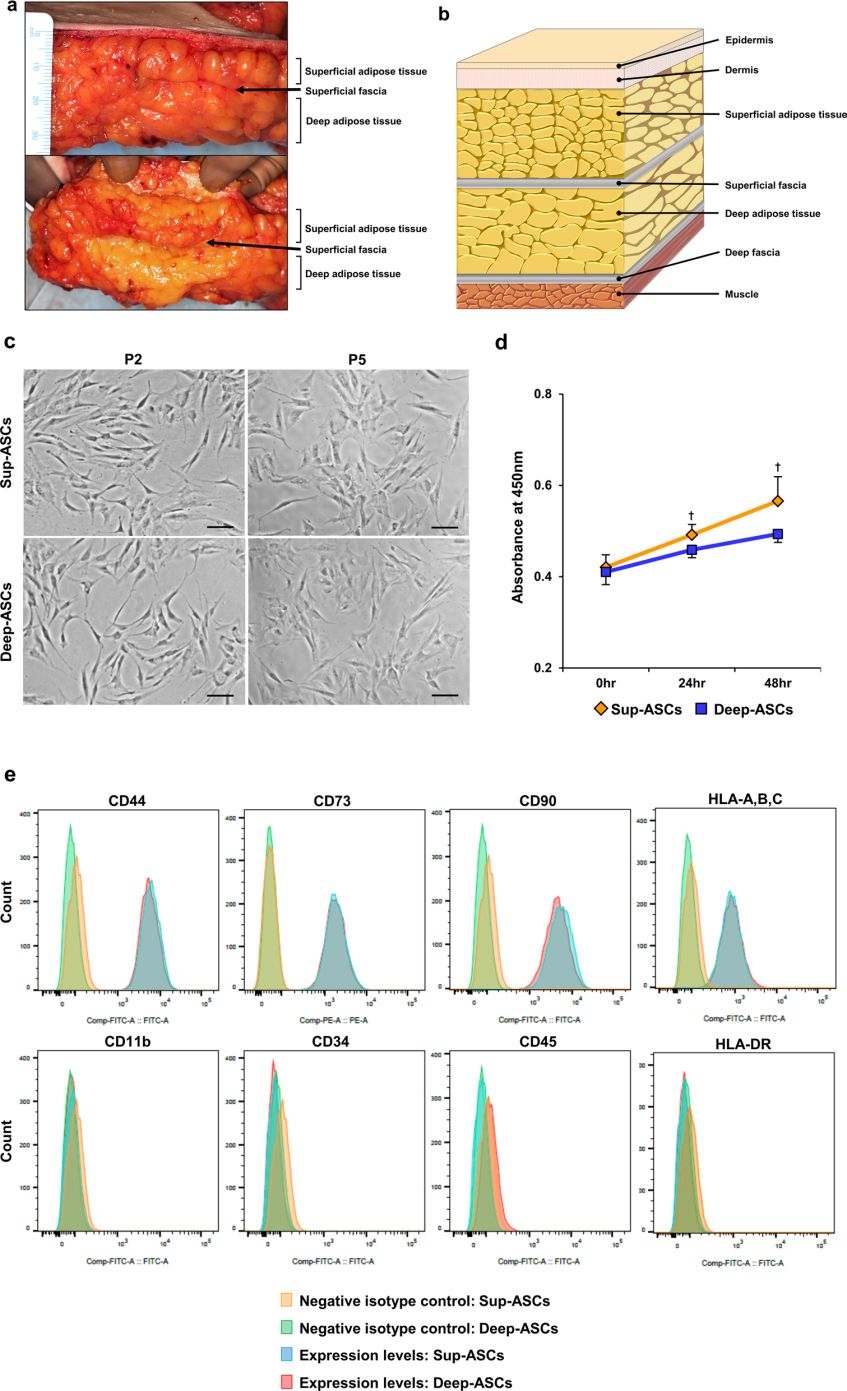

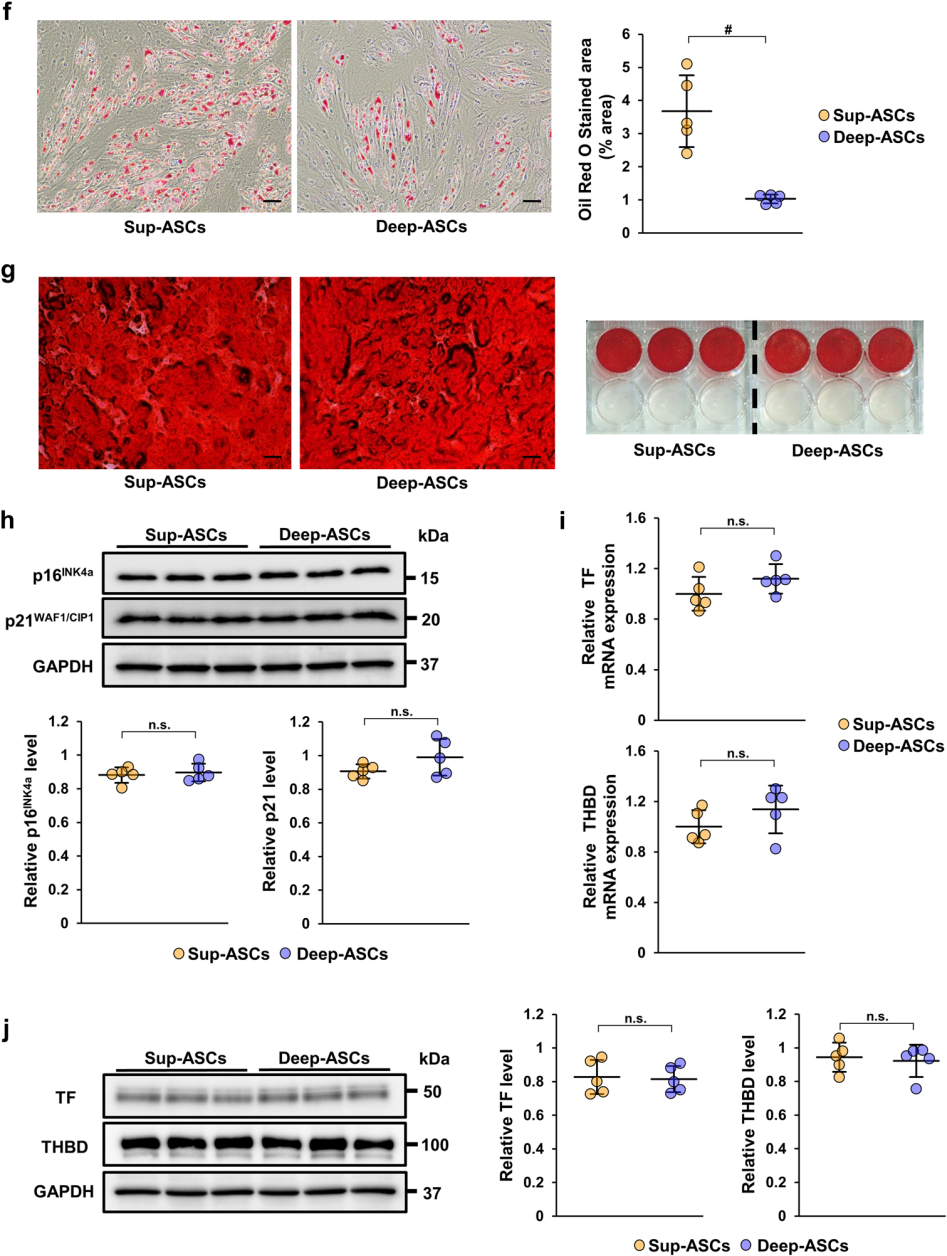
Characteristics of Sup-ASCs and Deep-ASCs. **a** Representative images of superficial and deep subcutaneous abdominal adipose tissues and superficial fascia. **b** Schematic of abdominal subcutaneous structures. **c** Representative images of Sup-ASCs and Deep-ASCs at passages 2 and 5 (scale bar = 100 μm). **d** Surviving cells were examined by a water-soluble tetrazolium salt (WST)-1 assay. Graph shows the absorbance value at each time point (0, 24, and 48 h) (n = 5 in each group). ^†^*P* < 0.05 versus Deep-ASCs. **e** Flow cytometric analysis of surface marker expression on Sup-ASCs and Deep-ASCs. **f, g** Representative images of Sup-ASCs and Deep-ASCs after staining with Oil Red O and Alizarin Red S (scale bar = 100 μm). Graph shows quantification of the Oil Red O-stained area as percentages of the total area (n = 5 in each group). **h** Western blot analysis of p16^INK4a^ and p21^WAF1/CIP1^ in Sup-ASCs and Deep-ASCs. Graphs show densitometric analyses of p16^INK4a^ and p21^WAF1/CIP1^ expression levels normalized to the GAPDH expression level (n = 5 in each group). Full-length blots are presented in Additional file 1A: Fig. 1a. **i** Expression levels of tissue factor (TF) and thrombomodulin (THBD) mRNAs in Sup-ASCs and Deep-ASCs (n = 5 in each group). **j** Western blot analysis of TF and THBD in Sup-ASCs and Deep-ASCs. Graphs show densitometric analyses of TF and THBD expression levels normalized to the GAPDH expression level (n = 5 in each group). Full-length blots are presented in Additional file 1A: Fig. 1b. Data are means ± S.D. ^#^*P* < 0.01; n.s.: not significant (Student’s t-test)

MSCs can be isolated from various tissues such as adipose tissue, bone marrow, placenta, and umbilical cord [17, 18]. Among them, subcutaneous abdominal adipose tissue can be obtained by liposuction with a small incision and contains a high amount of adipose-derived MSCs (ASCs) [19, 20]. However, as shown in Fig. 1a, b, subcutaneous abdominal adipose tissue is divided into superficial and deep layers by a superficial fascia. Superficial adipose tissue contains small and tightly packed fat lobules [21, 22], whereas deep adipose tissue contains large and loosely packed fat lobules [21, 22]. Adipose tissue easily collected by liposuction is usually obtained from a deep layer [23, 24]. Therefore, ASCs derived from a deep layer are generally used for regenerative medicine. ASCs retain memory of functional heterogeneity in each adipose tissue from which the ASCs were isolated [25], raising the possibility that ASCs derived from superficial and deep subcutaneous adipose tissues (Sup-ASCs and Deep-ASCs, respectively) have different functions. So far, although many studies have reported that ASCs exert beneficial therapeutic effects [26–28], no research has been conducted to investigate differences in the therapeutic effects of Sup-ASCs and Deep-ASCs. Therefore, it has been unknown whether Deep-ASCs collected by abdominal liposuction are suitable for an intravascular ASC therapy of organ injury compared with Sup-ASCs.

To our best knowledge, it is unclear whether there is a difference in the therapeutic effects on organ injury between Sup-ASCs and Deep-ASCs. An ischemia–reperfusion injury (IRI) model, in which organ injury induces chronic inflammation resulting in fibrosis, is suitable to evaluate the therapeutic effects of ASCs on organ injury [15, 29]. Therefore, we compared the therapeutic efficacies of Sup-ASCs and Deep-ASCs using a renal IRI model.

## Methods

### Preparation of ASCs

ASCs were isolated from superficial and deep subcutaneous abdominal adipose tissues collected from patients (age range, 44–57 years) who underwent breast reconstruction, herein referred to as “Sup-ASCs” and “Deep-ASCs,” respectively. These cells were cultured in low glucose Dulbecco’s modified Eagle’s medium (DMEM; Sigma-Aldrich, St. Louis, MO) containing 10% FBS (Sigma-Aldrich) and were used in all experiments up to five passages. The Medical Ethics Committee of Hiroshima Graduate School of Biomedical Science permitted collection of the adipose tissue (Permit number: E-1516, registered on January 29, 2019). Each patient had provided written informed consent.

### Flow cytometric analysis

Flow cytometric analysis was performed in accordance with previously described methods [15]. An anti-human CD44 IgG antibody (BioLegend, San Diego, CA, 338804, 1:20), anti-human CD73 IgG antibody (BioLegend, 344004, 1:20), anti-human CD90 IgG antibody (BioLegend, 328108, 1:20), anti-human CD11b IgG antibody (BioLegend, 301404, 1:20), anti-human CD34 IgG antibody (BioLegend, 343504, 1:20), anti-human CD45 IgG antibody (BioLegend, 304006, 1:20), anti-human HLA-A–C IgG antibody (BioLegend, 311404, 1:20), and anti-human HLA-DR IgG antibody (BioLegend, 307604, 1:20) were used. Stained ASCs were analyzed by a BD FACSVerse (Becton, Dickinson and Company, Franklin Lakes, NJ). The data were evaluated by FlowJo software (FlowJo, LLC, Ashland, OR).

### Cell proliferation assay

Proliferative activity of ASCs was examined by a water-soluble tetrazolium salt (WST)-1 assay (Takara Bio, Shiga, Japan). ASCs (5 × 10^3^/100 μL) were seeded in 96-well microplates and cultured in DMEM containing 10% FBS. After incubation for 0, 24, and 48 h, 10 μL WST-1 reagent was added to each well, followed by culture for 2 h. Absorbance was measured using a microplate reader at a wavelength of 450 nm and reference wavelength of 620 nm.

### Differentiation experiments

ASCs were cultured in adipogenic differentiation medium (Takara Bio) or osteogenic differentiation medium (Sigma-Aldrich) for 14 days in accordance with the manufacturers’ protocols. Oil Red O (Sigma-Aldrich) and Alizarin Red S (FUJIFILM Wako Pure Chemical, Osaka, Japan) were used to assess adipogenic and osteogenic differentiation, respectively. The stained area was estimated using ImageJ software by examining five randomly selected fields.

### Preparation of conditioned medium

To generate conditioned medium from Sup-ASCs and Deep-ASCs (Sup-ASC-CM and Deep-ASC-CM, respectively), ASCs (5 × 10^5^/dish) were seeded in 10 cm dishes and incubated in DMEM containing 10% FBS. At 80% confluence, the culture medium was replaced with DMEM containing 0.1% FBS, followed by culture for 24 or 48 h. Then, each medium was collected.

### Enzyme-linked immunosorbent assay (ELISA)

ELISAs of prostaglandin E2 (PGE2) (Enzo Life Science, Farmingdale, NY), vascular endothelial growth factor (VEGF) (R&D Systems, Minneapolis, MN), and hepatocyte growth factor (HGF) (R&D Systems) were performed in accordance with the manufacturers’ protocols. Concentrations were normalized to the total protein content.

### Cell culture and treatments

HK-2 cells were purchased from the American Type Culture Collection (Manassas, VA) and cultured in accordance with a previously described method [15]. Cells at 70% confluence were exposed to serum starvation in DMEM containing 0.1% FBS or conditioned medium from ASCs for 24 h, and then 10 ng/ml recombinant human transforming growth factor (TGF)-β1 (R&D Systems) was added to the cells directly. HK-2 cells were collected 30 min after the addition of TGF-β1 (to examine the p-Smad2 protein levels) and 24 h after the addition of TGF-β1 (to investigate α-SMA protein levels), and then used for in vitro experiments.

THP-1 cells were also purchased from the American Type Culture Collection and cultured following previously described methods [29]. To induce differentiation of THP-1 cells into macrophages, THP-1 cells were exposed to 160 nM phorbol 12-myristate 13-acetate (Sigma-Aldrich) for 48 h. Subsequently, the medium was changed to conditioned medium from ASCs. After 24 h, the cells were collected and subjected to in vitro experiments.

### Animals

Male Sprague Dawley (SD) rats (8 weeks old) for IRI model establishment were obtained from Charles River Laboratories Japan (Yokohama, Japan). A total of 20 male SD rats were used in this study. All rats were reared in standard cages under a 12-h light–dark cycle at approximately 25 °C and 40–60% humidity, and were provided with free access to food and water. They were randomly divided into four groups (n = 5 in each group): sham, PBS, Sup-ASC, and Deep-ASC groups. All animal experimental procedures were approved by the Institutional Animal Care and Use Committee of Hiroshima University (Hiroshima, Japan) (Permit number: A16-83) and conducted following the “Guide for the Care and Use of Laboratory Animals, 8th ed, 2010” (National Institutes of Health, Bethesda, MD). The study results were reported in accordance with ARRIVE guidelines 2.0.

### Experimental animal model

Renal IRI was induced by transiently clamping the unilateral renal artery. Rats were anesthetized by an intraperitoneal injection of three types of mixed anesthetic agents: medetomidine (0.15 mg/kg), midazolam (2 mg/kg), and butorphanol (2.5 mg/kg). After performing a laparotomy, the left kidney was exposed. Subsequently, the renal pedicle was clamped by atraumatic vascular clamps for 1 h, followed by reperfusion on a heating blanket. After reperfusion, PBS only or ASCs (2.5 × 10^5^ cells/rat) in 0.2 ml PBS were administered through the abdominal aorta clamped above and below the left renal artery bifurcation. At 21 days post-injection, the rats were sacrificed by exsanguination under anesthesia (medetomidine 0.15 mg/kg, midazolam 2 mg/kg, butorphanol 2.5 mg/kg, intraperitoneally) and their left kidneys were collected to assess fibrosis.

### Western blot analysis

Sample collection and western blotting were carried out following previously described methods [15]. A rabbit monoclonal anti-p16^INK4a^ antibody (Abcam, Cambridge, UK, ab51243, 1:2500), rabbit monoclonal anti-p21^WAF1/CIP1^ antibody (Abcam, ab109199, 1:1000), rabbit monoclonal anti-tissue factor (TF) antibody (Abcam, ab228968, 1:1000), rabbit monoclonal anti-thrombomodulin (THBD) antibody (Abcam, ab109189, 1:5000), rabbit monoclonal anti-CD163 antibody (Abcam, ab182422, 1:1000), rabbit polyclonal anti-CD68 antibody (Abcam, ab125212, 1:1000), mouse monoclonal anti-α-SMA antibody (Sigma-Aldrich, A2547, 1:5000), mouse monoclonal anti-GAPDH antibody (Sigma-Aldrich, G8795, 1:5000), rabbit monoclonal anti-phosphorylated Smad2 (p-Smad2) antibody (Cell Signaling Technology, Danvers, MA, #3108, 1:1000), mouse monoclonal anti-Smad2 antibody (Cell Signaling Technology, #3103, 1:1000), and mouse monoclonal anti-α-tubulin antibody (Sigma-Aldrich, T9026, 1:5000) were used as primary antibodies. Horseradish peroxidase-conjugated goat anti-rabbit immunoglobulin G (Dako, Glostrup, Denmark) or goat anti-mouse immunoglobulin G (Dako) were used as secondary antibodies. SuperSignal West Dura or Pico system (Thermo Fisher Scientific, Rockford, IL) was used to detect signals. The intensity of each band was quantified by ImageJ software (version 1.47v; National Institutes of Health) and standardized to the level of either GAPDH or α-tubulin.

### Histological and immunohistochemical analyses

Sections of formalin-fixed, paraffin-embedded tissues were stained with hematoxylin and eosin (HE) and Masson trichrome for morphological and fibrotic evaluation in accordance with previously described protocols [15]. Areas of interstitial fibrosis were evaluated using a Lumina Vision (Mitani, Osaka, Japan) by examining five randomly selected fields (× 200) of the cortex. Immunohistochemical staining was also performed in accordance with previously described methods [15]. A mouse monoclonal anti-α-SMA antibody (Sigma-Aldrich, A2547, 1:5000) was used as the primary antibody. The positive area was estimated using ImageJ software by examining five randomly selected fields (× 200) of the cortex.

### Quantitative real-time reverse transcription-PCR

RNA extraction and real-time reverse-transcription PCR were performed in accordance with previously described methods [15]. Specific oligonucleotide primers and probes for human TF (assay ID: Hs00175225_m1), human THBD (assay ID: Hs00264920_s1), human tumor necrosis factor-α-induced protein 6 (TSG-6) (assay ID: Hs00200180_m1), rat collagen type I (assay ID: Rn01463848_m1), rat collagen type III (assay ID: Rn01437681_m1), human β-actin (assay ID: Hs99999903_m1), and rat GAPDH (assay ID: Rn01775763_g1) were obtained as TaqMan Gene Expression Assays (Applied Biosystems, Foster City, CA). mRNA levels were normalized to the mRNA level of either β-actin or GAPDH.

### RNA-sequencing

RNA extraction from ASCs was performed following previously described methods [15]. Three biological replicates were prepared for each ASC type. RNA-sequencing was performed by Macrogen Japan (Tokyo, Japan). The total RNA concentration was calculated by Quant-IT RiboGreen (Invitrogen, Waltham, MA). To assess the integrity of total RNA, samples are run on the TapeStation RNA screentape (Agilent, Santa Clara, CA). Only high-quality RNA preparations with RIN < 7.0 were used for RNA library construction. A library was independently prepared with 1 µg total RNA for each sample using the Illumina TruSeq Stranded mRNA Sample Prep Kit (Illumina, Inc., San Diego, CA). The qualified libraries were sequenced using an Illumina NovaSeq (Illumina, Inc.) with paired-end (2 × 100 bp) reads. After raw reads were trimmed and quality controlled, the processed reads were aligned to *Homo sapiens* (hg38) using HISAT v2.1.0 [30]. Then, transcript assembly of known transcripts was processed by StringTie v1.3.4d [31, 32]. On the basis of the results, expression abundances of transcripts and genes were calculated as the read count or fragments per kilobase of exon per million mapped read value per sample.

### Enrichment analysis of differentially expressed genes

Transcripts with fold-change values > 2.0 with *P* ≤ 0.05 were included in the analysis as differentially expressed genes (DEGs). Gene Ontology (GO) functional enrichment analysis of the significant gene list was performed using g:Profiler tool (https://biit.cs.ut.ee/gprofiler/).

### Statistical analysis

All experiments were performed three times and similar results were obtained. Results are expressed as means ± standard deviations (S.D.). For multiple group comparisons, one-way ANOVA followed by Tukey–Kramer’s post-hoc test was applied. Comparisons between two groups were analyzed by Student’s t-test. *P* < 0.05 was considered statistically significant.

## Results

### Comparison of Sup-ASC and Deep-ASC characteristics

Similar numbers of ASCs were obtained from 10 g of superficial and deep subcutaneous abdominal adipose tissue, respectively. We first compared the MSC characteristics of Sup-ASCs and Deep-ASCs. They exhibited comparable typical spindle shapes (Fig. 1c). After repeated passaging, they displayed a comparable senescence-like morphology such as enlarged cell bodies and irregular shapes (Fig. 1c). WST-1 assays showed that Sup-ASCs had a stronger proliferative ability than Deep-ASCs (Fig. 1d). Flow cytometry revealed that both ASC types similarly expressed standard MSC markers, such as CD44, CD73, CD90, and HLA-A–C, and did not express MSC-negative markers such as CD11b, CD34, CD45, and HLA-DR (Fig. 1e). Additionally, we compared the capacity of both ASC types to differentiate into adipocytes and osteoblasts. As shown in Fig. 1f, Oil Red O staining indicated that Sup-ASCs had a stronger ability to differentiate into adipocytes compared with Deep-ASCs. Moreover, Alizarin Red S revealed no significant difference in the osteogenic differentiation capacity between Sup-ASCs and Deep-ASCs (Fig. 1g). Subsequently, we compared cell senescence of Sup-ASCs and Deep-ASCs at passage 5. p16^INK4a^ and p21^WAF1/CIP1^ protein levels were almost equivalent between both ASC types (Fig. 1h, Additional file 1A: Fig. S1a). Several studies have reported that ASCs exert a procoagulant effect with evidence indicating that TF, which is expressed on the ASC surface, plays a pivotal role in this effect [33, 34]. Therefore, we compared TF expression between Sup-ASCs and Deep-ASCs. We found no significant difference in the protein and mRNA levels of TF between them (Fig. 1i, j, Additional file 1A: Fig. S1b). Moreover, the protein and mRNA levels of THBD, which is an anticoagulant factor, were almost equal in Sup-ASCs and Deep-ASCs (Fig. 1i, j, Additional file 1A: Fig. S1b).

### Sup-ASCs and Deep-ASCs equally induce a change in the macrophage phenotype to immunosuppressive M2

**Fig. 2:**
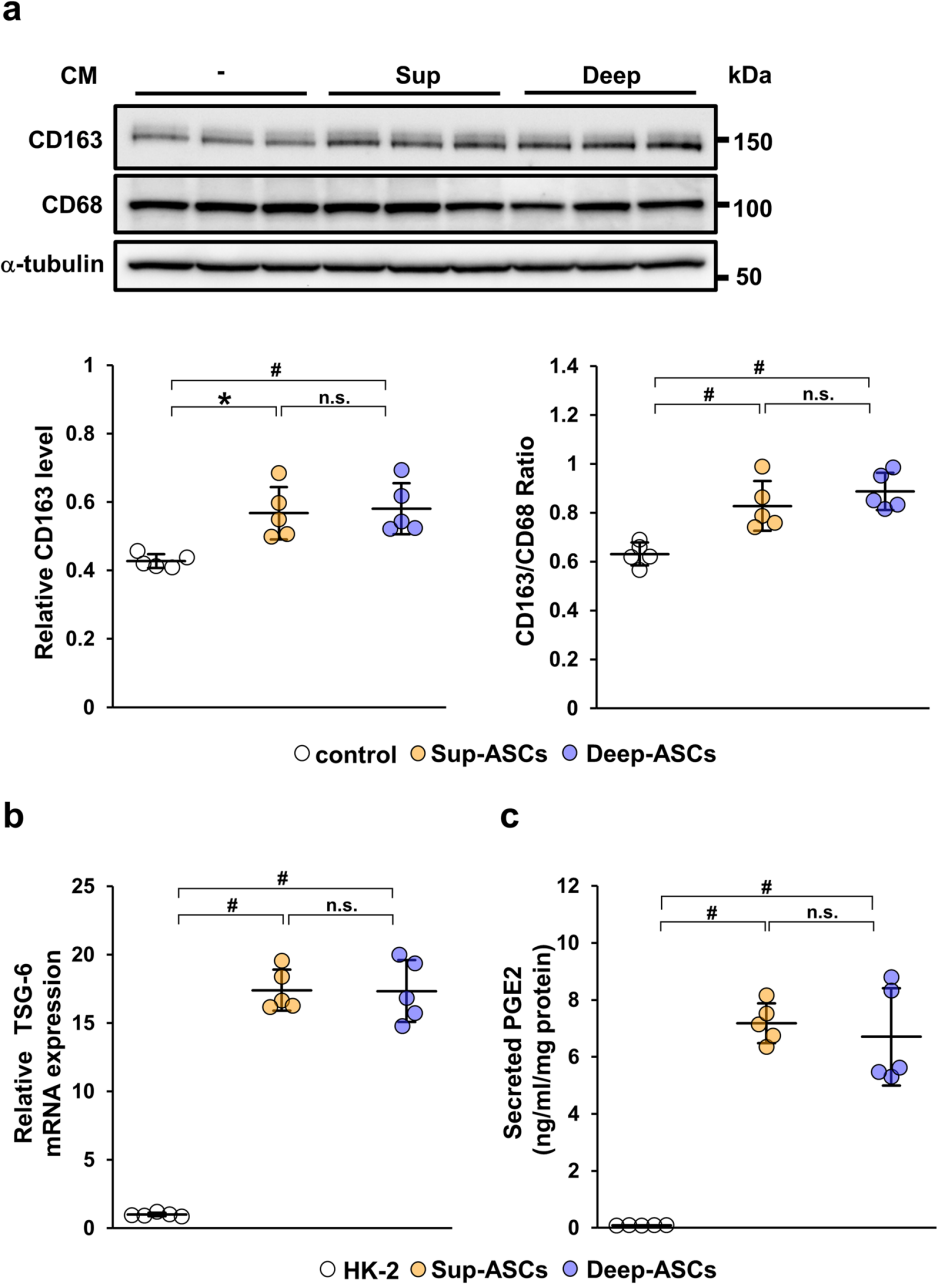
Sup-ASC-CM and Deep-ASC-CM equivalently induce a change in the macrophage phenotype to M2. **a** Western blot analysis of CD163 and CD68 in THP-1 macrophages treated with Sup-ASC-CM or Deep-ASC-CM. Graph shows densitometric analysis of the CD163 expression level normalized to CD68 and α-tubulin expression levels (n = 5 in each group). Full-length blots are presented in Additional file 2B: Fig. 1c. **b** Expression level of TSG-6 mRNA in Sup-ASCs and Deep-ASCs (n = 5 in each group). **c** Concentrations of prostaglandin E2 (PGE2) in Sup-ASC-CM and Deep-ASC-CM were measured by an ELISA (n = 5 in each group). Data are means ± S.D. ^#^*P* < 0.01, *P < 0.05; n.s.: not significant (one-way ANOVA followed by Tukey–Kramer’s post-hoc test)

MSCs induce a phenotypic change in macrophages to immunosuppressive M2 by releasing TSG-6 and PGE2, resulting in anti-inflammatory effects [35, 36]. To assess whether there was a difference in such induction of M2 macrophages between Sup-ASCs and Deep-ASCs, we examined CD163 expression in THP-1 cells cultured in Sup-ASC-CM or Deep-ASC-CM. Although the CD163 protein level was upregulated by both ASC-CMs, there was no significant difference between Sup-ASC-CM and Deep-ASC-CM (Fig. 2a, Additional file 1B: Fig. S1c). Furthermore, we compared the TSG-6 mRNA expression level in Sup-ASCs and Deep-ASCs. As shown in Fig. 2b, no significant difference in the TSG-6 mRNA level was observed between them. We also compared the PGE2 concentrations in Sup-ASC-CM and Deep-ASC-CM using an ELISA. No significant difference in the PGE2 protein level was found between them (Fig. 2c).

### Conditioned media from Sup-ASCs and Deep-ASCs equally suppress fibrotic changes through inhibition of TGF-β/Smad signaling

**Fig. 3:**
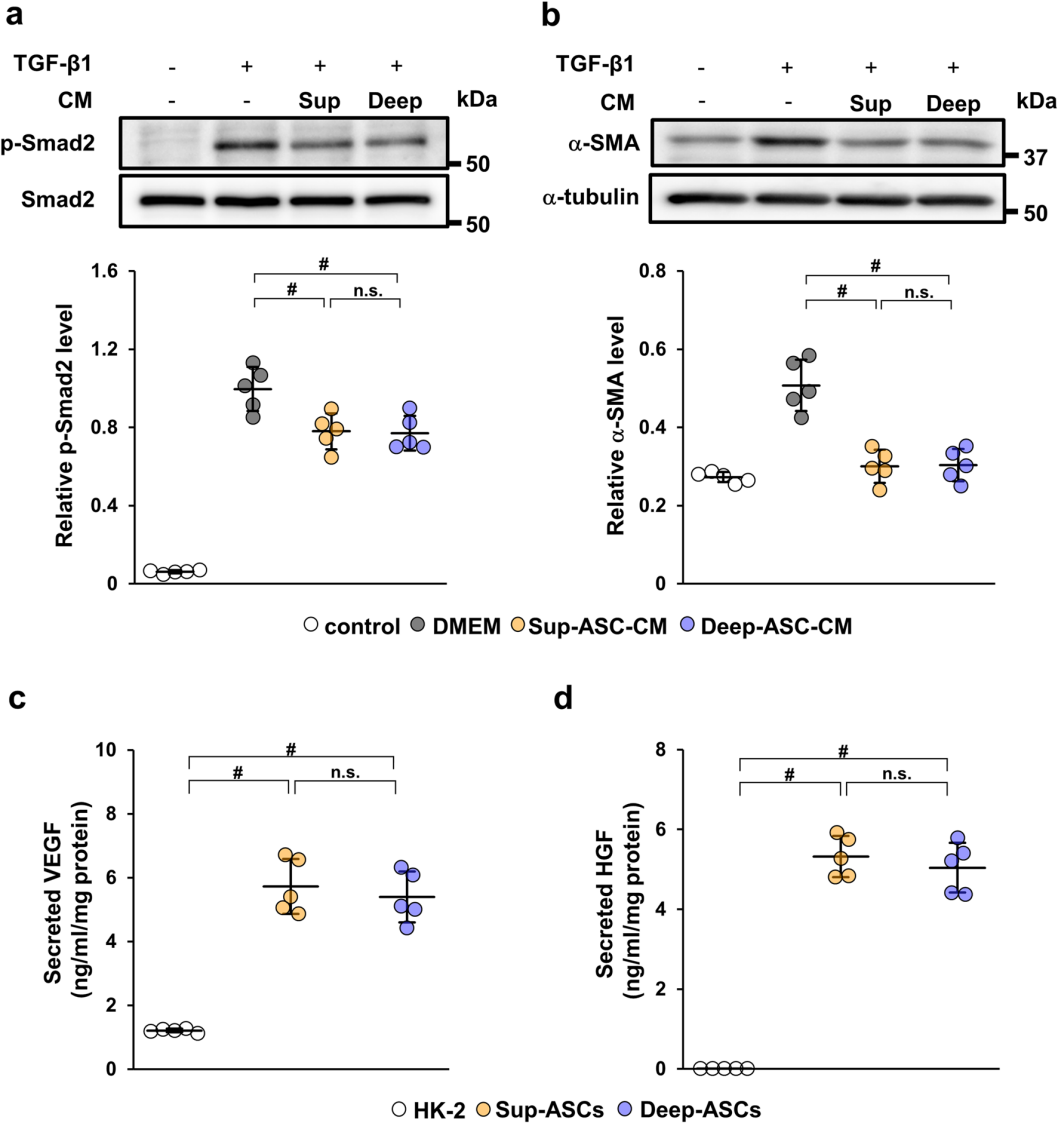
Sup-ASC-CM and Deep-ASC-CM equally suppress fibrotic changes through inhibition of TGF-β/Smad signaling in HK-2 cells. **a** Western blot analysis of phosphorylated Smad2 (p-Smad2) in HK-2 cells stimulated with TGF-β1 for 30 min. Graph shows densitometric analysis of the p-Smad2 expression level normalized to the Smad2 expression level (n = 5 in each group). Full-length blots are presented in Additional file 2B: Fig. 1d. **b** Western blot analysis of α-SMA in HK-2 cells stimulated with TGF-β1 for 24 h. Graph shows densitometric analysis of the α-SMA expression level normalized to the α-tubulin expression level (n = 5 in each group). Full-length blots are presented in Additional file 3C: Fig. 1e. **c, d** Concentrations of vascular endothelial growth factor (VEGF) and hepatocyte growth factor (HGF) in Sup-ASC-CM and Deep-ASC-CM were measured by ELISAs (n = 5 in each group). Data are means ± S.D. ^#^*P* < 0.01; n.s.: not significant (one-way ANOVA followed by Tukey–Kramer’s post-hoc test)

The TGF-β/Smad signaling pathway is considered to play a central role in fibrosis progression [10, 11]. To investigate a difference in the direct inhibitory effect on TGF-β/Smad signaling between Sup-ASCs and Deep-ASCs, we examined the expression of p-Smad2 and α-SMA in TGF-β1-stimulated HK-2 cells treated with Sup-ASC-CM or Deep-ASC-CM. The p-Smad2 protein level was upregulated by TGF-β1 stimulation, which was significantly reduced by treatments with both Sup-ASC-CM and Deep-ASC-CM with no significant difference between them (Fig. 3a, Additional file 1B: Fig. S1d). Similar results were obtained for α-SMA protein expression (Fig. 3b, Additional file 1C: Fig. S1e). Additionally, we measured the concentrations of VEGF and HGF, which are implicated in the anti-fibrotic effects of ASCs [15, 29, 37], in Sup-ASC-CM and Deep-ASC-CM. We found no significant difference in VEGF and HGF protein levels between them (Fig. 3c, d).

### Sup-ASCs and Deep-ASCs equally ameliorate IRI-induced renal fibrosis in rats

**Fig. 4:**
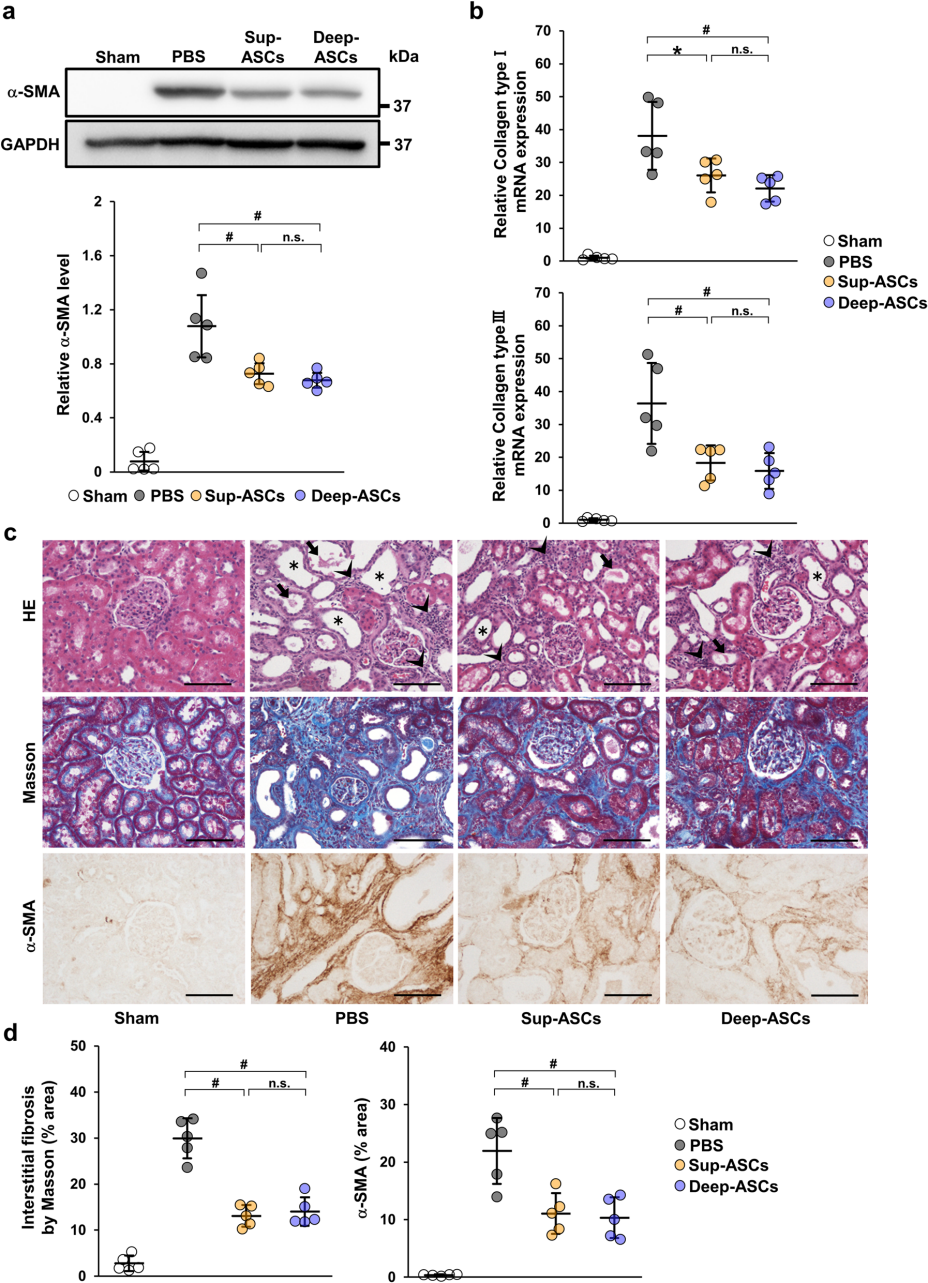
Sup-ASCs and Deep-ASCs equally attenuate renal fibrosis in IRI rats. **a** Western blot analysis of α-SMA in the kidney cortex of IRI rats at day 21 post-IRI. Graph shows densitometric analyses of α-SMA expression levels normalized to the GAPDH expression level (n = 5 in each group). Full-length blots are presented in Additional file 3C: Fig. 1f. **b** Expression levels of collagen type I and III mRNAs in the kidney cortex of IRI rats at day 21 post-IRI (n = 5 in each group).** c** Representative images of immunohistochemical staining of α-SMA as well as HE and Masson trichrome staining of kidney sections at day 21 post-IRI (scale bar = 200 μm). HE staining shows tubular dilatation (asterisk), tubular cast formation (arrow), and inflammatory cells (head arrow).** d** Quantification of the interstitial fibrosis area and α-SMA-positive area as percentages of the total area (n = 5 in each group). Data are means ± S.D. ^#^*P* < 0.01, *P < 0.05; n.s.: not significant (one-way ANOVA followed by Tukey–Kramer’s post-hoc test)

To assess whether there was a difference in the therapeutic effect on renal fibrosis between Sup-ASCs and Deep-ASCs, we investigated α-SMA expression in the IRI model that had been administered PBS, Sup-ASCs, or Deep-ASCs. As shown in Fig. 4a, the α-SMA protein level was markedly increased in IRI rats injected with PBS (PBS group). Although this upregulated expression was significantly suppressed in IRI rats injected with Sup-ASCs (Sup-ASC group) and Deep-ASCs (Deep-ASC group), no significant difference was observed between the groups (Fig. 4a, Additional file 1C: Fig. S1f). Similarly, collagen type I and III mRNA levels were decreased in Sup-ASC and Deep-ASC groups with no significant difference between them (Fig. 4b). Furthermore, HE staining showed tubular dilatation, tubular cast formation, and diffused infiltration of inflammatory cells in the PBS group. These tubulointerstitial injuries were equally attenuated in Sup-ASC and Deep-ASC groups (Fig. 4c). Masson trichrome staining revealed that the area of interstitial fibrosis was significantly reduced in both ASC groups with no significant difference between them (Fig. 4c, d). Similarly, immunostaining showed that the α-SMA-positive area was equivalently reduced in Sup-ASC and Deep-ASC groups (Fig. 4c, d).

### RNA-sequencing analysis of Sup-ASCs and Deep-ASCs

**Fig. 5:**
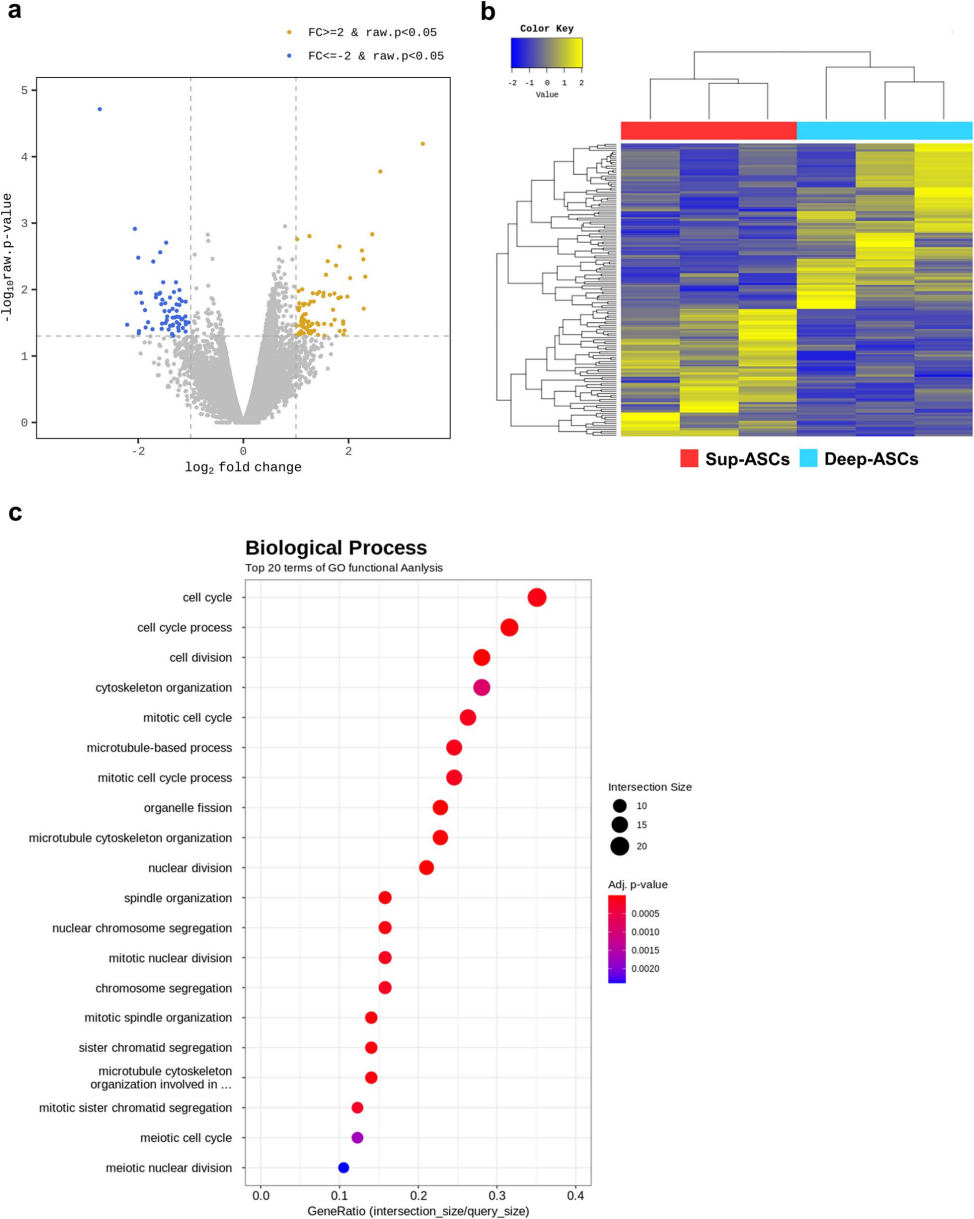
RNA-sequencing of Sup-ASCs and Deep-ASCs. **a** Volcano plots showing gene expression profiles of Sup-ASCs and Deep-ASCs (n = 3 in each group). Yellow and blue points represent upregulated (FC ≥ 2, *P* < 0.05) and downregulated (FC ≤  − 2.0, *P* < 0.05) differentially expressed genes (DEGs), respectively. Gray points represent genes with no significant differences in expression. **b** Hierarchical clustered heat map showing the gene expression patterns of DEGs (FC ≤  − 2.0, or ≥ 2.0, *P* < 0.05) between Sup-ASCs and Deep-ASCs (n = 3 in each group). Yellow represents upregulation of gene expression and blue represents downregulation of gene expression. **c** Gene Ontology enrichment analysis of DEGs by comparing Sup-ASCs with Deep-ASCs (n = 3 in each group)

To identify differences between Sup-ASCs and Deep-ASCs in more detail, we performed RNA-sequencing. Volcano plots revealed that, out of the 18,377 quantitatively detected genes, 147 DEGs were identified between both ASC types, including 83 significantly upregulated DEGs and 64 significantly downregulated DEGs, when comparing Sup-ASCs with Deep-ASCs (Fig. 5a). A heat map of the DEGs prepared by hierarchical clustering revealed clear differences in mRNA expression between the ASC types (Fig. 5b). Additionally, to investigate the function of these DEGs, we performed GO functional enrichment analysis. We found significant differences in the expression of genes involved in cell proliferation in Sup-ASCs compared with that in Deep-ASCs, whereas no significant difference was found in the expression of genes related to their anti-inflammatory and anti-fibrotic effects (Fig. 5c).

## Discussion

**Fig. 6:**
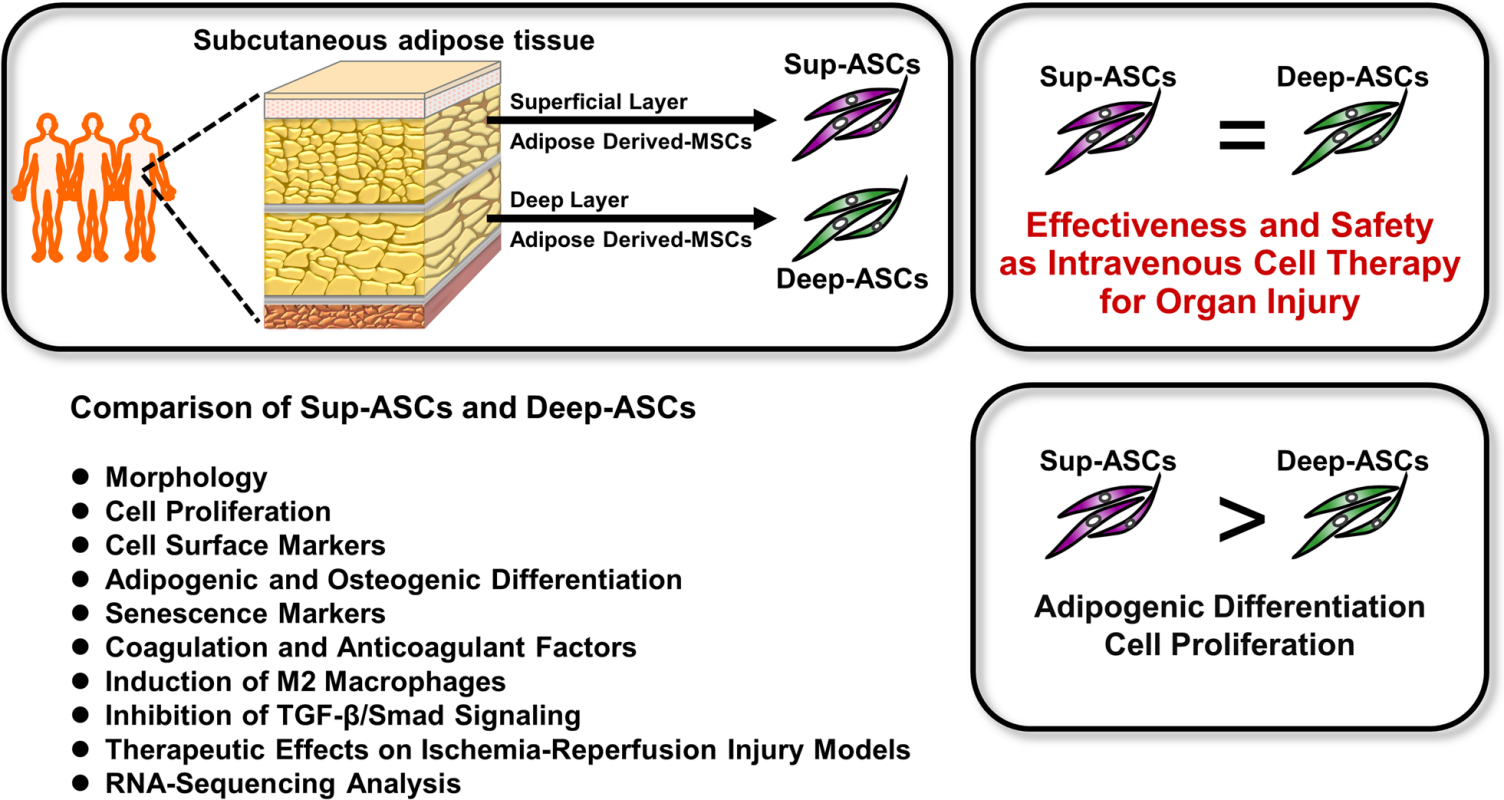
Comparison of Sup-ASCs and Deep-ASCs. As intravenous cell therapy for organ injury, treatment efficacy and safety are comparable between Sup-ASCs and Deep-ASCs, whereas Sup-ASCs have a stronger ability to proliferate and differentiate into adipocytes compared with Deep-ASCs

Our study demonstrated that the cell characteristics, including morphology, cell surface markers, cell senescence markers, and expression of coagulation and anticoagulant factors, were almost equivalent between Sup-ASCs and Deep-ASCs, whereas Sup-ASCs had a stronger ability to proliferate and differentiate into adipocytes compared with Deep-ASCs. Furthermore, there was no significant difference in the ability to promote polarization of M2 macrophages and inhibit TGF-β1-induced fibrotic changes between them. In the in vivo experiments, administration of Sup-ASCs and Deep-ASCs equally attenuated renal fibrosis induced by IRI in rats. Additionally, RNA-sequencing analysis revealed no significant difference in the expression of genes involved in anti-inflammatory and anti-fibrotic effects between Sup-ASCs and Deep-ASCs. These results indicate that both Sup-ASCs and Deep-ASCs can be used effectively and safely as an intravascular ASC therapy for organ injury (Fig. 6).

Several studies have revealed that adipose-derived ASCs isolated from different anatomical locations have different biological functions [38, 39]. This functional diversity of ASCs is attributed to their origin, cellular context, and surrounding microenvironment [38, 39]. This fact led us to hypothesize that Sup-ASCs and Deep-ASCs have different therapeutic effects on organ injury. Moreover, several studies have shown that infrared light has positive effects on MSCs, including enhanced cell proliferation [40, 41] and migration [40, 42]. Additionally, a reduced culture temperature decreases reactive oxidative species and apoptosis in cells [43, 44]. Considering that Sup-ASCs are located near the body exterior, they are more likely to be influenced by these factors compared with Deep-ASCs. Therefore, we initially expected that Sup-ASCs would have stronger therapeutic effects than Deep-ASCs. However, in contrast to our expectations, we observed no significant difference in the therapeutic potencies of both ASC types.

MSCs were previously considered to promote regeneration of injured tissue by their ability to differentiate into multiple tissue types [45]. However, recently, rather than differentiating, paracrine activity has been thought of as a major mechanism that contributes to tissue regeneration [12, 13]. MSCs secrete various humoral factors, and notably, PGE2, VEGF, and HGF play pivotal roles in tissue repair [15, 29, 36, 37]. Our study revealed that the capacity to secrete these factors was also similar between Sup-ASCs and Deep-ASCs as well as therapeutic potency. Meanwhile, MSCs have a procoagulant activity, which raises the concern of a thrombogenic risk [33, 34]. Several studies have reported that the procoagulant activity induced by MSCs is associated with the TF expression level [33, 34]. In this study, the TF expression level was comparable between Sup-ASCs and Deep-ASCs, suggesting that the procoagulant activity is also comparable between Sup-ASCs and Deep-ASCs.

RNA-sequencing is used to comprehensively analyze the transcriptome, and as such, it is a very useful method for discovering differences in biological functions between two cell types. In this study, RNA-sequencing was performed to investigate differences between Sup-ASCs and Deep-ASCs in more detail. Genes involved in biological processes that had a higher expression in Sup-ASCs were related to the cell cycle, cell division, or nuclear division, as part of the machinery involved with self-renewal, and included *CENPF* (2.72-fold), *CENPE* (2.24-fold), *BUB1B* (2.23-fold), and *ANLN* (2.09-fold). This result suggests that Sup-ASCs have higher proliferative capacity compared with Deep-ASCs, which is consistent with the result of the WST-1 assay. MSCs were reported to secrete various inflammatory factors [46]. Thus, we checked the DEG list against cytokine activity (GO: 0005125). Although we found only *PF4* in the list, there were no other genes related to inflammatory factors. Moreover, we did not identify any GO terms involved with inflammation in the GO functional enrichment analysis. Therefore, we think that Sup-ASCs and Deep-ASCs secrete similar inflammatory factors.

We previously reported the localization and maintenance of engrafted MSCs injected to IRI rats [47]. In that study, we administered 5.0 × 10^5^ DiI-labeled MSCs from human bone marrow using the same method as in the current study. Engrafted MSCs were clearly detected in the IRI kidney for up to 21 days and were sparsely detected by 42 days post-IRI. Furthermore, we confirmed that 5.0 × 10^5^ MSCs from bone marrow had a significant therapeutic benefit [15, 36, 47]. In the current study, we initially administered 5.0 × 10^5^ ASCs as in our previous study. However, 2 of 10 rats developed renal infarction after the administration of ASCs. Several studies reported that ASCs had stronger immunomodulatory effects than MSCs derived from bone marrow [48, 49], while ASCs had greater procoagulant effects [34]. Because we were concerned about the possibility of blood clot formation induced by ASCs, we administered ASCs at half the previous dose (2.5 × 10^5^) in this study.

## Conclusions

We showed that cell characteristics, except for cell proliferation and adipogenic differentiation, are comparable between Sup-ASCs and Deep-ASCs. Moreover, Sup-ASCs and Deep-ASCs not only have comparable therapeutic potencies but also similar expression of coagulation and anticoagulant factors. These findings suggest that Deep-ASCs obtained by abdominal liposuction can be used effectively and safely as an intravascular ASC therapy of organ injury.

## Supplementary Information

**Additional file 1: Fig. S1**. Full-length western blot images. a Full-length blot images for Fig. 1h. b Full-length blot images for Fig. 1j

**Additional file 2: Fig. S1**. Full-length western blot images. c Full-length blot images for Fig. 2a. d Full-length blot images for Fig. 3a

**Additional file 3: Fig. S1**. Full-length western blot images. e Full-length blot images for Fig. 3b. f Full-length blot images for Fig. 4a

## Data Availability

The data that support the findings of this study are available from the corresponding author upon reasonable request. RNA sequencing data reported in this paper are available in the DNA Data Bank of Japan (DDBJ) Sequenced Read Archive (https://www.ddbj.nig.ac.jp/dra/index.html) under the accession numbers DRA014406. Gene expression data are deposited in the DDBJ Genomic Expression Archive (https://www.ddbj.nig.ac.jp/gea/index.html) under the accession numbers E-GEAD-506. The data is available at the following URL: https://ddbj.nig.ac.jp/public/ddbj_database/gea/experiment/E-GEAD-000/E-GEAD-506/.

## Abbreviations

DEGs: Differentially expressed genes
DMEM: Dulbecco’s modified Eagle’s medium
ELISA: Enzyme-linked immunosorbent assay
FBS: Fetal bovine serum
GO: Gene Ontology
HE: Hematoxylin and eosin
HGF: Hepatocyte growth factor
IRI: Ischemia–reperfusion injury
MSCs: Mesenchymal stem cells
Sup-ASCs: MSCs derived from superficial subcutaneous adipose tissue
Deep-ASCs: MSCs derived from deep subcutaneous adipose tissue
Sup-ASC-CM: Conditioned medium from Sup-ASCs
Deep-ASC-CM: Conditioned medium from Deep-ASCs
PBS group: IRI rats injected with PBS
Sup-ASC group: IRI rats injected with Sup-ASCs
Deep-ASC group: IRI rats injected with Deep-ASCs
PGE2: Prostaglandin E2
p-Smad2: Phosphorylated Smad2
S.D.: Standard deviations
TF: Tissue factor
THBD: Thrombomodulin
TSG-6: Tumor necrosis factor-α-induced protein 6
TGF: Transforming growth factor
VEGF: Vascular endothelial growth factor
WST: Water-soluble tetrazolium salts
