# Supplementary figures and images for: Comparison of therapeutic effects of mesenchymal stem cells derived from superficial and deep subcutaneous adipose tissues
Source: Stem Cell Res Ther. 2023 May 4;14:121. doi: 10.1186/s13287-023-03350-3 (PMC10161523; doi:10.1186/s13287-023-03350-3)

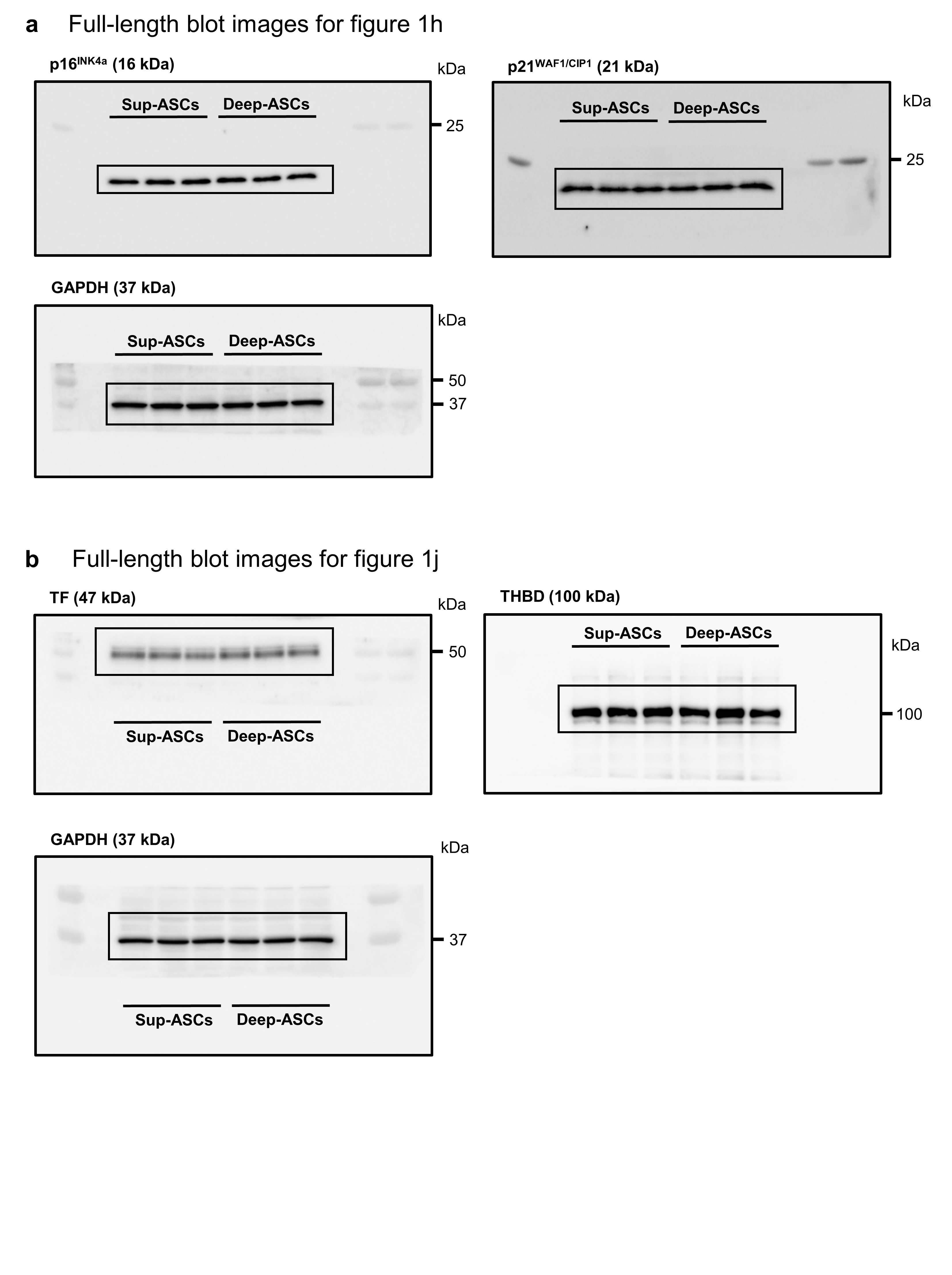

Supplement: Supplementary file 1 — Additional file 1: Fig. S1. Full-length western blot images. a Full-length blot images for Fig. 1h. b Full-length blot images for Fig. 1j [file 13287_2023_3350_MOESM1_ESM.tif]

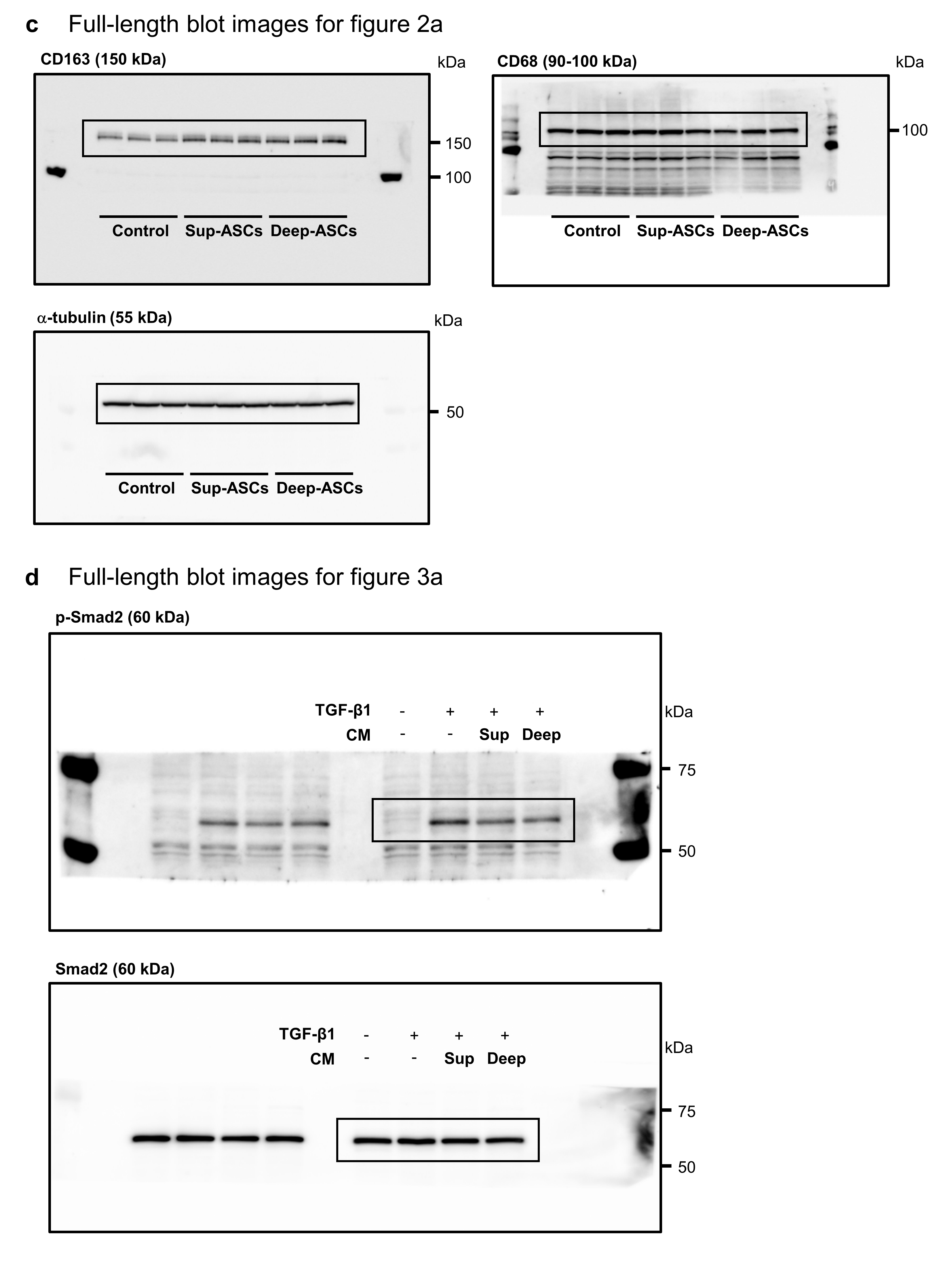

Supplement: Supplementary file 2 — Additional file 2: Fig. S1. Full-length western blot images. c Full-length blot images for Fig. 2a. d Full-length blot images for Fig. 3a [file 13287_2023_3350_MOESM2_ESM.tif]

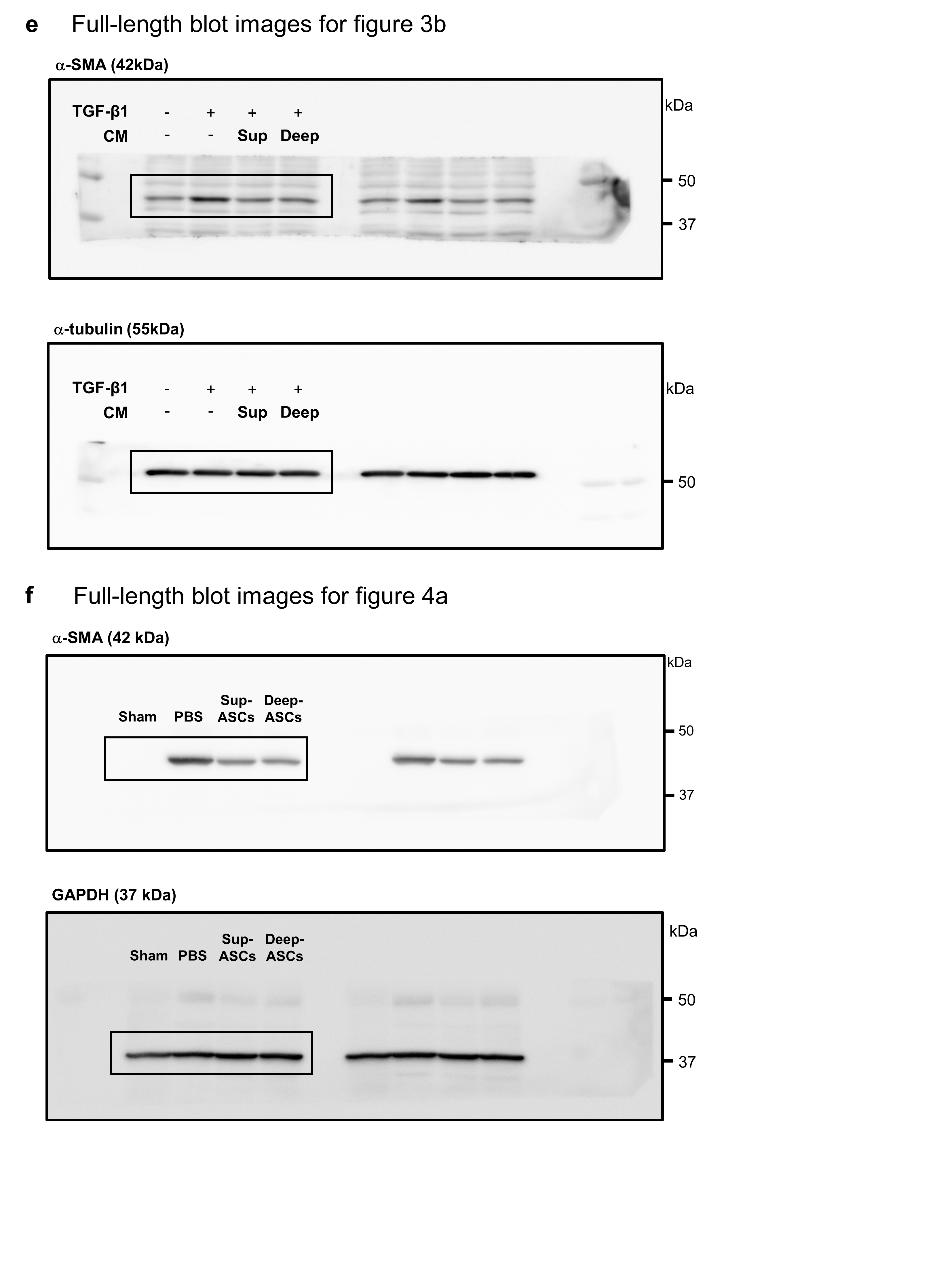

Supplement: Supplementary file 3 — Additional file 3: Fig. S1. Full-length western blot images. e Full-length blot images for Fig. 3b. f Full-length blot images for Fig. 4a [file 13287_2023_3350_MOESM3_ESM.tif]
